# Supplementary material for: UPLC-MS Analysis, Quantification of Compounds, and Comparison of Bioactivity of Methanol Extract and Its Fractions from Qiai (Artemisia argyi Lévl. et Van.)
Source: Molecules. 2023 Feb 21;28(5):2022. doi: 10.3390/molecules28052022 (PMC10004512; doi:10.3390/molecules28052022)
Supplement: Supplementary file 1 [file molecules-28-02022-s001.zip › molecules-2216587-supplementary.pdf]

Table S1. The optimized MRM parameters of the fourteen target analytes.

Table S2. Antioxidant potential of extract and fractions of QA evaluated by DPPH and ABTS method.

Table S3. Inhibition of NO release by LPS-stimulated Raw264.7 cells of extract and fractions of QA

Table S4. Diameter of inhibition zone of the extract and fractions of QA

Figure S1. Bacterial inhibition of *P. vulgaris* (A), *B.subtilis* (B), *S.aureus* (C), *E.coli* (D), *P.aeruginosa* (E) by samples in Disc diffusion assay.

Table S1. The optimized MRM parameters of the fourteen target analytes.

NO	Analyte	RT	Monitoring ion	Transitions (amu)	Fragmentor (V)	Collision energy (V)
1	Chlorogenic acid	4.99	$[M-H]^-$	353.10→137.06	32	44
2	Neochlorogenic acid	3.76	[M-H]-	353.10→191.09	44	18
3	4-Dicaffeoylquinic acid	5.24	[M-H]-	353.00→173.08	38	16
4	3,5-di-O-caffeoylquinic acid	9.87	[M-H]-	515.10→191.09	18	30
5	3,4-di-O-caffeoylquinic acid	9.22	[M-H]-	515.10→173.08	56	28
6	4,5-di-O-caffeoylquinic acid	11.07	[M-H]-	515.10→353.12	56	18
7	Hyperoside	8.25	[M-H]-	463.03→300.19	40	24
8	Chrysoeriol 7-O-glucoside	11.10	[M-H]-	461.10→255.08	74	44
9	Chrysoeriol	13.68	[M-H]-	299.03→22.13	64	30
10	Schaftoside	7.18	[M-H]-	563.10→383.13	90	32
11	Isoschaftoside	7.68	[M-H]-	563.10→443.07	80	28
12	Hispidulin	16.16	[M-H]-	299.03→137.06	54	30
13	Jaceosidin	16.73	[M-H]-	329.10→314.10	42	18
14	Eupatilin	19.31	[M-H]-	343.10→313.08	44	26

Table S2. Antioxidant potential of extract and fractions of QA evaluated by DPPH and ABTS method.

Samples	DPPH	ABTS
	IC50 value (μg/mL)	IC50 value (μg/mL)
QA-TE	119.87 ± 1.32	543.25 ± 4.87
QA-FPE	/	/
QA-FEA	303.33 ± 3.51	601.33 ± 5.19
QA-FWT	58.34 ± 0.79	270.87 ± 2.36
Trolox	11.79 ± 0.27	220.06 ± 0.78

DPPH, 2.2-dy-phenyl-1-picrylhydrazyl; ABTS, 2,2′-Azino-bis (3-ethylbenzothiazoline-6-sulphonic acid); Trolox,6-hydroxy-2,5,7,8-tetramethylchroman-2-Carboxylic acid; data are presented as means ± SD, n = 3.

Table S3. Inhibition of NO release by LPS-stimulated Raw264.7 cells of extract and fractions of QA

Samples	Concentrations(μg/ml)	NO (μM/L)	%Inhibition
QA-TE	5	13.08±0.12	1.22±0.91
	10	11.71±0.13	11.56±0.95
	15	11.34±0.08	14.35±0.58
	20	10.64±0.11	19.64±0.84
	25	8.35±0.03	36.93±0.20
QA-FPE	5	13.15±0.05	0.68±0.34
	10	12.97±0.10	2.04±0.77
	15	12.06±0.14	8.91±1.08
	20	11.42±0.09	13.75±0.72
	25	10.10±0.09	23.72±0.67
QA-FEA	5	9.71±0.06	26.66±0.42
	10	7.28±0.05	45.02±0.36
	15	6.36±0.05	51.96±0.36
	20	5.22±0.04	60.57±0.31
	25	4.00±0.10	69.79±0.79
QA-FWT	5	12.13±0.09	8.38±0.68
	10	10.08±0.13	23.87±1.02
	15	8.77±0.19	33.76±1.46
	20	5.93±0.21	55.40±1.58
	25	5.34±0.11	59.67±0.83

Data are presented as means ± SD, n = 4.

Table S4. Diameter of inhibition zone of the extract and fractions of QA

Microorganisms	QA-TE	QA-FPE	QA-FEA	QA-FWT	CH
P.vulgaris	17.3±1.2	13.7±0.6	16.3±1.5	17.7±0.6	30.3±1.5
B.subtilis	11.7±0.6	10.7±0.6	13.3±0.6	20.3±2.1	33.9±1.5
S.aureus	20.7±0.6	14.0±1.0	18.7±0.6	22.3±0.6	25.7±0.6
E.coli	16.7±0.6	14.7±0.6	16.7±0.6	20.0±1.0	33.7±1.2
P.aeruginosa	14.7±1.5	12.7±0.6	15.7±0.6	18.7±1.2	32.3±1.5

Data are presented as means ± SD, n = 3.

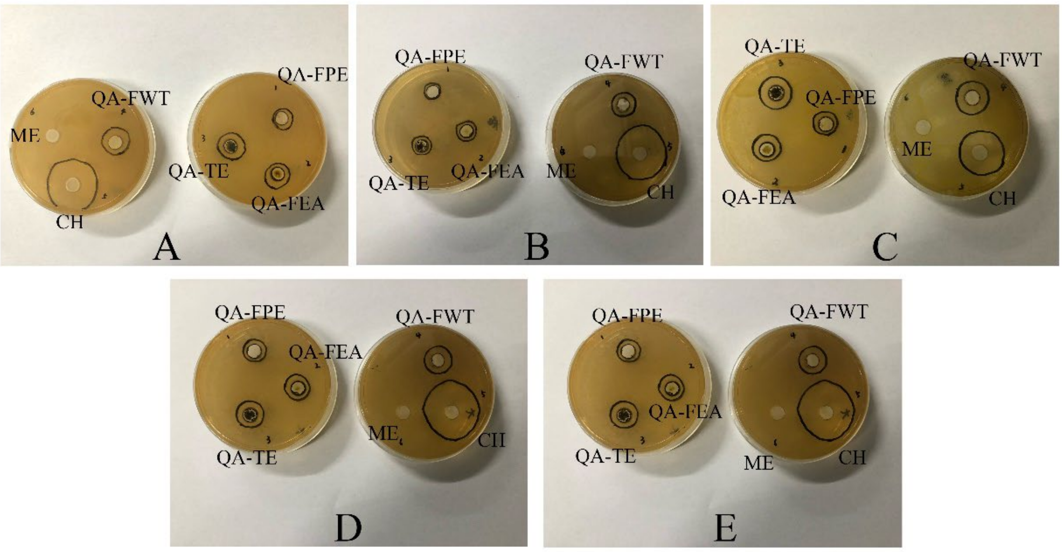

Figure S1. Bacterial inhibition of *P. vulgaris* (A), *B.subtilis* (B), *S.aureus* (C), *E.coli* (D), *P.aeruginosa* (E) by samples in Disc diffusion assay.
